# Zika Brazilian Cohorts (ZBC) Consortium: Protocol for an Individual Participant Data Meta-Analysis of Congenital Zika Syndrome after Maternal Exposure during Pregnancy

**DOI:** 10.3390/v13040687

**Published:** 2021-04-16

**Authors:** Maria das Graças Costa Alecrim, Melania Maria Ramos de Amorim, Thalia Velho Barreto de Araújo, Patrícia Brasil, Elizabeth B. Brickley, Marcia da Costa Castilho, Bernadete Perez Coelho, Antônio José Ledo Alves da Cunha, Geraldo Duarte, Cássia Fernanda Estofolete, Ricardo Queiroz Gurgel, Juliana Herrero-Silva, Cristina Barroso Hofer, Aline Siqueira Alves Lopes, Celina Maria Turchi Martelli, Adriana Suely de Oliveira Melo, Demócrito de Barros Miranda-Filho, Ulisses Ramos Montarroyos, Maria Elisabeth Moreira, Marisa Marcia Mussi-Pinhata, Consuelo Silva de Oliveira, Saulo Duarte Passos, Arnaldo Prata-Barbosa, Darci Neves dos Santos, Lavínia Schuler-Faccini, Antônio Augusto Moura da Silva, Isadora Cristina de Siqueira, Patrícia da Silva Sousa, Marília Dalva Turchi, Ricardo Arraes de Alencar Ximenes, Ana Laura de Sene Amâncio Zara, Zika Brazilian Cohorts Consortium (ZBC-Consortium)

**Affiliations:** 1 Fundação de Medicina Tropical Doutor Heitor Vieira Dourado, Manaus 69040-000, Brazil; galecrim.br@gmail.com (M.d.G.C.A.); mycastilho13@gmail.com (M.d.C.C.); 2 Universidade Federal de Campina Grande, Campina Grande 58428-830, Brazil; profmelania.amorim@gmail.com (M.M.R.d.A.); asomelo@gmail.com (A.S.d.O.M.); 3 Instituto de Medicina Integral Professor Fernando Figueira (IMIP), Recife 50070-902, Brazil; 4 Departamento de Medicina Social, Universidade Federal de Pernambuco, Recife 50670-901, Brazil; thalia.araujo@ufpe.br; 5 Instituto Nacional de Infectologia Evandro Chagas, Fundação Oswaldo Cruz, Rio de Janeiro 21040-360, Brazil; patricia.brasil@ini.fiocruz.br; 6 London School of Hygiene & Tropical Medicine, London WC1E 7HT, UK; elizabeth.brickley@lshtm.ac.uk; 7 Centro de Ciências da Saúde, Universidade Federal de Pernambuco, Recife 50670-901, Brazil; bernadeteperez@uol.com.br; 8 Departamento de Pediatria, Universidade Federal do Rio de Janeiro, Rio de Janeiro 21941-971, Brazil; antonioledo@yahoo.com.br (A.J.L.A.d.C.); cbhofer@hucff.ufrj.br (C.B.H.); 9 Faculdade de Medicina de Ribeirão Preto, Universidade de São Paulo, Ribeirão Preto 14049-900, Brazil; gduarte@fmrp.usp.br (G.D.); mmmpinha@fmrp.usp.br (M.M.M.-P.); 10 Faculdade de Medicina de São José do Rio Preto, São José do Rio Preto 15090-000, Brazil; cassiafestofolete@gmail.com; 11 Universidade Federal de Sergipe, Aracaju 49060-108, Brazil; ricardoqgurgel@gmail.com (R.Q.G.); aline.siq@icloud.com (A.S.A.L.); 12 Secretaria Municipal de Tangará da Serra, Tangará da Serra 78300-000, Brazil; julianaherrerodasilva@hotmail.com; 13 Instituto Aggeu Magalhães, Fundação Oswaldo Cruz, Recife 50740-465, Brazil; turchicm@gmail.com; 14 Instituto Paraibano de Diagnóstico (EMBRION), Campina Grande 58400-506, Brazil; 15 Faculdade de Ciências Médicas, Universidade de Pernambuco, Recife 50100-130, Brazil; demofilho@gmail.com; 16 Instituto de Ciências Biológicas, Universidade de Pernambuco, Recife 50100-130, Brazil; ulisses.montarroyos@upe.br; 17 Instituto Fernandes Figueira, Fundação Oswaldo Cruz, Rio de Janeiro 22250-020, Brazil; bebethiff@gmail.com; 18 Instituto Evandro Chagas, Ananindeua 67030-000, Brazil; consuelooliveira@iec.gov.br; 19 Faculdade de Medicina de Jundiaí, Jundiaí 13211-383, Brazil; sauloduarte@uol.com.br; 20 Instituto D’Or de Pesquisa e Ensino, Rio de Janeiro 22281-100, Brazil; arnaldoprata@globo.com; 21 Instituto Saúde Coletiva, Universidade Federal da Bahia, Salvador 40110-040, Brazil; darci@ufba.br; 22 Universidade Federal do Rio Grande do Sul, Porto Alegre 91501-970, Brazil; lavinia.faccini@ufrgs.br; 23 Departamento de Saúde Pública, Universidade Federal do Maranhão, São Luís 65020-070, Brazil; aamouradasilva@gmail.com; 24 Centro de Pesquisas Gonçalo Moniz, Salvador 40296-710, Brazil; isadora.siqueira@fiocruz.br; 25 Centro de Referência em Neurodesenvolvimento, Assistência e Reabilitação de Crianças, Secretaria de Saúde do Estado do Maranhão, São Luís 65076-820, Brazil; cdneuropatricia@gmail.com; 26 Departamento de Saude Coletiva, Universidade Federal de Goiás, Goiânia 74605-050, Brazil; marilia.turchi@gmail.com (M.D.T.); analaurazara@ufg.br (A.L.d.S.A.Z.); 27 Departamento de Medicina Tropical da Universidade Federal de Pernambuco, Recife 50670-901, Brazil

**Keywords:** cohort, Zika, pregnant women, microcephaly, congenital Zika syndrome, IPD meta-analysis

## Abstract

Despite great advances in our knowledge of the consequences of Zika virus to human health, many questions remain unanswered, and results are often inconsistent. The small sample size of individual studies has limited inference about the spectrum of congenital Zika manifestations and the prognosis of affected children. The Brazilian Zika Cohorts Consortium addresses these limitations by bringing together and harmonizing epidemiological data from a series of prospective cohort studies of pregnant women with rash and of children with microcephaly and/or other manifestations of congenital Zika. The objective is to estimate the absolute risk of congenital Zika manifestations and to characterize the full spectrum and natural history of the manifestations of congenital Zika in children with and without microcephaly. This protocol describes the assembly of the Consortium and protocol for the Individual Participant Data Meta-analyses (IPD Meta-analyses). The findings will address knowledge gaps and inform public policies related to Zika virus. The large harmonized dataset and joint analyses will facilitate more precise estimates of the absolute risk of congenital Zika manifestations among Zika virus-infected pregnancies and more complete descriptions of its full spectrum, including rare manifestations. It will enable sensitivity analyses using different definitions of exposure and outcomes, and the investigation of the sources of heterogeneity between studies and regions.

## 1. Introduction

Between August and October 2015, the Brazilian Ministry of Health identified a notable increase in microcephaly cases, initially in the state of Pernambuco. The largest number of cases was registered in this state, followed by the states of Paraíba and Bahia. On 10 November 2015, the Ministry of Health declared a Public Health Emergency of National Importance, and in February 2016, the World Health Organization declared a Public Health Emergency of International Concern, which was maintained until November of the same year.

Since the onset of the microcephaly epidemic, there has been intense mobilization by the academic community and national and international institutions, resulting in major advances in understanding of the human health consequences of intrauterine and postnatal exposure to Zika virus (ZIKV) infection. Investigations in Brazil provided the initial characterization of Congenital Zika Syndrome (CZS) [1,2,3] and the first clinical descriptions of microcephaly and the associated central nervous system (CNS) imaging [4,5] and of ocular manifestations [6] (later detailed in Ventura et al. [7,8,9], Freitas et al. [10]). In the area of laboratory diagnosis, researchers delineated the role of IgM in cerebrospinal fluid as a diagnostic tool [11] and developed an algorithm for integrating longitudinal molecular and serological assay results for defining ZIKV infections in pregnant women with rash [12]. In Pernambuco state, findings from the first microcephaly case control study contributed key evidence supporting the causal role of ZIKV in the microcephaly epidemic [13,14]. In parallel, the Rio de Janeiro cohort of pregnant women provided the first assessment of CZS risk after intrauterine exposure and reported heightened risks at early stages of gestation [15,16].

Despite advances made, many key epidemiological questions remain unanswered, and results between studies have demonstrated wide variation in the estimates of the absolute risk of congenital Zika manifestations following confirmed maternal ZIKV infection during pregnancy. For example, in the study by Brasil et al. [16], adverse pregnancy outcomes were found in 46% of the 125 pregnancies of women positive for ZIKV, while 49 of the 117 live births (42%) had abnormalities by physical examination and/or imaging. In the study by Rice et al. [17], 203 children (14%) born to 1450 RT-PCR-positive pregnant women in the United States were born with a Zika-associated birth defect and/or a neurodevelopmental abnormality possibly due to Zika virus infection. Hoen et al. [18] found a frequency of neurological and ocular changes possibly associated with ZIKV infection in 39 (7.0%) fetuses and children born to 546 pregnant women with a confirmed RT-PCR diagnosis residing in the French territories of the Americas. Though the difference with the study of Rice et al. [17] should be interpreted with caution as it is based on surveillance data, the differences between the two prospective cohorts are even more marked.

The reasons for this heterogeneity may be explained by regional or biological differences between study populations as well as by variations in methodological approaches. For instance, local variability in the mosquito endemicity, public health control efforts, pre-existing immunity, and population density may alter the course of the epidemic and the number of individuals infected but would not modify the risk of adverse outcomes in women already infected. On the other hand, methodological differences in exposure and outcome case definitions as well as variation in assessment techniques and instruments may have occurred. Differences in risk estimates between studies may also have arisen due to low precision due to small samples sizes of individual investigations. It is also worth mentioning that, due to the relatively small samples of the cohorts of pregnant women, it is likely that some of the rare outcomes were missed in individual studies. The Zika Brazilian Cohorts (ZBC) Consortium (ZBC Consortium) was assembled to begin to overcome these challenges and to improve understanding of the impact of ZIKV infections in pregnancy. 

The ZBC Consortium complements two other planned joint analyses of ZIKV infections in pregnancy: the World Health Organization-led Zika Virus Individual Participant Data Consortium [19] and the joint analysis of the prospective cohort studies of the European Commission-funded Zika Preparedness Latin American Network (ZikaPLAN), ZIKAlliance and ZIKAction consortia [20]. Though there is some overlap in the cohorts included in the three planned analyses, there are also important distinctions in the represented populations and analytical approaches. The ZBC Consortium differs from the others because of the relatively large sample size of some of the included cohort studies and because of the large number of individuals from the same country (i.e., Brazil) that are being included. These features will facilitate better exploration of within- and between-study heterogeneity and to compare subgroups of children with different characteristics who were evaluated using similar clinical approaches. The large sample size will also allow us to obtain more precise estimates and to study more adequately rare events. 

Since the beginning of the microcephaly epidemic, there has been great concern in Brazil regarding the standardization of protocols and data collection instruments for studying congenital Zika manifestations. With the support of Pan American Health Organization/World Health Organization (PAHO/WHO), a meeting of Brazilian and Latin American researchers was held in March 2016 to consolidate efforts to harmonize study protocols and instruments. Further discussions with international investigators were also held during subsequent protocol harmonization meetings held in Mexico in June 2016 and Geneva in February 2017. In October 2017, the Brazilian Ministry of Health and its Secretaries of Science, Technology, and Strategic Inputs (Secretaria de Ciência, Tecnologia e Insumos Estratégicos-SCTIE), Health Surveillance (Secretaria de Vigilância em Saúde-SVS) and Health Care (Sistema de Assistência à Saúde-SAS) hosted a “Zika-related Cohorts Consortium Meeting” in October 2017, which brought together Principal Investigators of studies at advanced stages of data collection from different regions of the country to initiate a new national partnership known as the Zika Brazilian Cohorts Consortium (ZBC Consortium). The early efforts of Brazilian researchers to harmonize cohort study protocols helped to make the resultant data more compatible, more alike, facilitating the planned pooled analysis.

The ZBC Consortium brings together and harmonizes epidemiological data from a series of prospective cohort studies, initiated during the 2015–2016 ZIKV epidemic, of pregnant women with rash and of children with microcephaly and/or other manifestations of congenital Zika. During four face-to-face meetings, principal investigators of the cohort studies have worked to: (1) elaborate a database management and authorship agreement; (2) identify common variables; (3) construct a data dictionary; (4) construct a harmonized dataset; (5) analyze the data; (6) discuss the results; and (7) prepare scientific articles. 

The data sharing and joint analyses of the ZBC Consortium will contribute to understanding of congenital Zika and will specifically enable a larger sample size and more robust estimates, an increased ability to quantify risks associated with rare events, and identification and quantification of sources of heterogeneity, if any exist.

## 2. ZBC Consortium Protocol

### 2.1. Objectives

The main objective of the ZBC Consortium is to jointly analyze data from Brazilian cohorts of pregnant women and children to obtain a more accurate estimation of the absolute risk of congenital Zika and a better characterization of the full spectrum of the syndrome among affected children with and without microcephaly. 

Two groups of prospective cohorts will be analyzed. The first group includes studies of pregnant women with rash (i.e., a common symptom of acute ZIKV infection) and will estimate the absolute risk of adverse outcomes compatible with exposure to ZIKV during pregnancy, by trimester at which infection occurred. The joint analysis of the cohorts of this group will be conducted in two steps. In the first step, we will estimate the absolute risk in children born to women who tested RT-PCR-positive for ZIKV during pregnancy for the following adverse outcomes: microcephaly, brain imaging abnormalities, neurological abnormalities and ophthalmological disorders. For the outcomes of microcephaly, brain imaging abnormalities, neurological abnormalities, and ophthalmological disorders, the absolute risk will be estimated for each outcome separately, for the presence of at least one of these outcomes, and, finally, for the concomitant presence of two or more of these abnormalities combined in different ways. This analysis will be performed using the findings at birth/first evaluation and, for the subset of children who were included in the children’s cohort, at any time during the follow-up. A subsequent analysis will be conducted to compare the relative risk of these outcomes in children born to women who tested RT-PCR-positive for ZIKV during pregnancy of the ZBC Consortium with the risk in children born to Brazilian unexposed mothers of the International Prospective Observational Cohort Study of Zika in Infants and Pregnancy (ZIP Study) [21]. This comparison will allow us to estimate the strength of the association and the excess risk of adverse outcomes in the exposed group compared to the unexposed.

The second group is composed of the cohorts of children with microcephaly and/or other adverse outcomes compatible with exposure to ZIKV and children born to mothers of the ZBC pregnant women cohorts, and its analysis aims to characterize the spectrum of clinical features, brain imaging abnormalities, ophthalmological disorders, neurological manifestations and neurocognitive development in these children. To characterize the full spectrum of congenital Zika manifestations, three groups of children will be compared: children with Zika-related microcephaly, children with other adverse outcomes compatible with exposure to ZIKV during pregnancy, and asymptomatic children born to women with laboratory evidence of ZIKV infection during pregnancy. A subsequent analysis will be carried out in which the ZIKV-exposed children of the ZBC Consortium will be compared with unexposed Brazilian children in the ZIP Study in relation to the type of abnormalities and their evolution, and in relation to the anthropometric measures and neurodevelopment.

Though the inclusion of the unexposed children of the ZIP study will provide a baseline-type comparison with a sample representative of the general population, there are some limitations in these comparisons. They are mainly related to differences in the forms and instruments to evaluate the children, the time gap in the beginning of the studies and the greater territorial coverage of the ZBC Consortium. In contrast to the ZIP study in which the teams who evaluated the children were blind to exposure status of the mothers and their offspring, for most of the cohorts of the ZBC Consortium, these teams knew that the children were born to women who presented symptoms compatible with Zika virus infection during pregnancy. To deal with the possibility of misclassification of the outcome, we will conduct a sensitivity analysis using different definitions. In relation to the potential difference in the frequency of abnormalities in children in the reference population of each cohort, we will also perform a sensitivity analysis restricted to exposed and unexposed children living in the same area. Concerning the time gap, it is not likely that the frequency of abnormal outcomes in the reference population would vary within this length of time. Finally, although there was a standardized protocol for the evaluation of children in cohorts, we cannot rule out that the children in the ZBC Consortium, being born to women who presented symptoms, were not more carefully investigated, which could lead to an overestimate in the difference between the exposed and unexposed groups.

### 2.2. Study Population, Eligibility and Inclusion Criteria

For the meta-analysis of the cohorts of pregnant women, studies were eligible to be included if they were based in Brazil and had recruited pregnant women with rash and laboratory evidence of acute ZIKV infection. Due to the potential for serological cross-reactivity, the main analysis will be restricted to pregnant women who tested positive for ZIKV by reverse transcription polymerase chain reaction (RT-PCR). Additional sensitivity analyses will be conducted including women who tested positive for ZIKV by other laboratory criteria, including: seroconversion during pregnancy (assessed at two time points by immunoglobulin (Ig) M, IgG, IgG3 or plaque reduction neutralization test (PRNT)) or symptoms (rash) plus at least one positive test result (IgM or PRNT) [12]. 

For the joint analysis of the children’s cohorts, Brazilian studies that had recruited children with congenital Zika manifestations or children born to pregnant women who had laboratory evidence of acute ZIKV infection as described above were considered eligible. Laboratory tests for ZIKV infection were not performed for all children and, for those who were tested, there was a variation in the biological specimen used, cerebrospinal fluid and blood samples being tested, respectively, for children with and without microcephaly. For these reasons and for the limitations of the diagnostic tests, children were categorized in groups according to different levels of evidence. Children with congenital Zika manifestations were classified into four categories: (i) definitive—with laboratory evidence of infection in the mother during pregnancy or in the newborn by serology, PRNT or RT-PCR, regardless of other findings; (ii) highly probable—imaging with specific features related to structural abnormalities in brain tissue, and negative laboratory testing for syphilis, toxoplasmosis and cytomegalovirus; (iii) moderately probable—imaging with specific features, but without results for one or more of the three infections (syphilis, toxoplasmosis and cytomegalovirus); (iv) somewhat probable cases—children for whom there was a report of imaging abnormality without detailed descriptions of findings but a physician of the research team concluded that a congenital infection was probably involved, and for whom laboratory results for syphilis, toxoplasmosis or cytomegalovirus were negative or unavailable [22]. Whereas somewhat probable cases were excluded from the primary analysis, moderately and highly probable cases were included because it has recently been shown that negative PRNT result does not exclude diagnosis of ZIKV infection [23]. The Microcephaly Epidemic Research Group (MERG) Pregnant Women’s Cohort showed that among ZIKV RT-PCR-positive mothers, less than half (48.5%), had a positive PRNT [12]. 

A subsequent analysis of the Pregnant Women Cohorts and of the Children Cohorts will include a comparison group of unexposed children from the ZIP Study who tested negative at birth and who were born to women who tested negative for ZIKV repeatedly (i.e., by RT-PCR and IgM) during pregnancy and until the 6th week after delivery [21]. Only the data of the Brazilian children and their mothers in this multi-country investigation will be analysed. These children underwent anthropometric, neurological and ophthalmologic analyses according to a standardised protocol and were followed up until the end of the first year of life.

### 2.3. Outcome Measures

The adverse outcomes assessed in the two meta-analyses will be the presence of microcephaly, brain imaging and neurological abnormalities, ophthalmological disorders, small for gestational age births, and stillbirth. Each of these summary outcomes was constructed based on the presence of specific clinical indicators and, in subsidiary analyses, we analyzed the risks of component adverse outcomes. For microcephaly, we will investigate both severity and proportionality. For neurological abnormalities, we will observe the presence of altered consciousness level/behaviour, inadequate visual or hearing response, localized motor deficit, abnormal tonus/trophism, sign of pyramidal release, and seizures. The brain imaging abnormalities will include the presence of calcification, ventriculomegaly, diffuse cortical atrophy, and other structural alterations. For ophthalmological disorders, we will consider the alterations of retina, optic nerve and fundus. 

### 2.4. Length of Follow-Up

Pregnant women were evaluated at least once during pregnancy. Their offspring were evaluated at least once at birth or in the first months after delivery. For the children included in the Children’s cohorts meta-analysis, the length of follow up varied in the different studies, with the longest period of follow-up being up to 4 years.

### 2.5. Sample Size

All Brazilian cohorts that met the inclusion criteria were included in the analysis independent of the sample size. As the full spectrum of manifestations compatible with ZIKV exposure during pregnancy is not known and some rare events may not have yet been identified, a larger sample size in the joint analysis will increase the chance to detect these events. In the meta-analysis, the option to perform a one-stage analysis was based on its suitability to work with rare events [24].

### 2.6. Study Identification, Data Sources, and Data Storage

**Figure 1:**
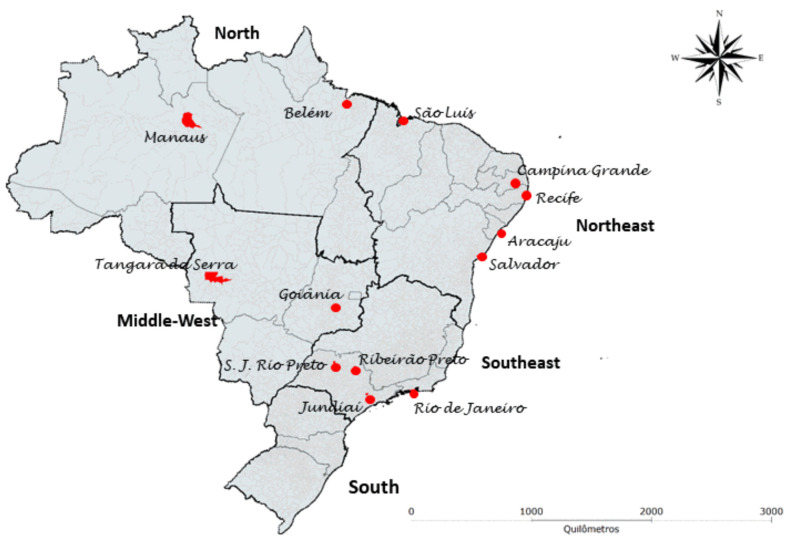
Municipalities with Brazilian cohorts of pregnant women and cohorts of children, participating in the ZBC Consortium.

The ZBC Consortium sought to identify all Brazilian cohorts of pregnant women with rash possibly associated with ZIKV infection and cohorts of children with microcephaly and/or other manifestations of congenital Zika. To this end, all groups that received funding from the Brazilian National Council for Scientific Research and Development (Conselho Nacional de Desenvolvimento Científico e Tecnológico-CNPq), the Coordination for the Improvement of Higher Level-Education Personnel (Coordenação de Aperfeiçoamento de Pessoal de Nível Superior - CAPES) and the Brazilian Ministry of Health were contacted regarding their research related to ZIKV infection. Those who participated in the harmonization meetings of research instruments and protocols hosted by PAHO/WHO in Recife (March 2016), Mexico (June 2016), and Geneva (February 2017) were also invited. All identified groups were asked to refer other cohort studies that were not on the list initially raised. In total, 12 cohorts of pregnant women and 15 cohorts of children followed by 16 research groups are included in the ZBC Consortium. The cohorts represent the North, Northeast, Middle-West and Southeast regions of Brazil (Figure 1).

Due to the participation of Brazilian groups in both the ZBC Consortium and the ZIP Study, it will be possible to include all Brazilian ZIP sites, i.e., Recife and Salvador in the Northeast, and Rio de Janeiro and Ribeirão Preto in the Southeast, in the analysis.

### 2.7. Extraction of Data

The same procedures were applied to collect individual participant data from the pregnant women and children’s cohorts. Participating cohorts provided all questionnaires and case report forms, which were searched to identify variables relevant to the proposed objectives and common to all studies. For the cohorts of pregnant women, variables providing the following information were selected: characteristics of pregnancy and infection; laboratory tests for diagnosis of ZIKV infection; birth outcomes: children’s head circumferences and anthropometric measurements, brain imaging abnormalities, morphological characteristics, and clinical, neurological, ophthalmological and hearing alterations. For the cohorts of children, in addition to the outcomes described above, variables related to neurocognitive development and electroencephalogram (EEG) were selected. Data related to repeated measures were collected to evaluate the evolution of the different manifestations of congenital Zika.

All the cohort databases were first standardized by each research team using specially developed data collection tools (i.e., a baseline questionnaire and a data dictionary (defining the formats and categorization of variables)), which facilitated the sharing of harmonizable databases. Then, in a secure “Shared Data Platform,” the databases of all associated cohorts were jointly stored. The creation of this platform and its management structures reflected the diversity and complexity of data from different sources and formats (e.g., imaging findings) that needed to be stored, analyzed and shared. The development software used for the platform was GeneXus X Ev 1, which develops web environment applications using aspx code. The Informatics Sector of the Aggeu Magalhães Institute/Fiocruz/Pernambuco designed and protected the Shared Data Platform. All data were anonymized and protected by password. Access was restricted to the ZBC Consortium coordinators. 

The ZIP Study dataset will be harmonized with the ZBC Consortium dataset following the same standardization. The ZIP Study dataset has most of the critical variables of the ZBC Consortium. Exposed and unexposed mothers and children were followed using the same standardized protocol except for transfontanelar ultrasound, which was performed mainly on the children born to exposed mothers. 

### 2.8. Analysis Plan

The ZBC Consortium Analysis Committee will synthesize the individual participant data from the two groups of cohort studies.

#### 2.8.1. Descriptive Analysis

Aggregate descriptions of the individual participants in the cohorts will be provided. For the cohorts of pregnant women and children, analyses will include descriptions of covariate frequencies and distributions. For quantitative variables, including: head circumference at beginning and at other moments of reevaluation; mean head circumference of children with microcephaly; mean head circumference of children with other anomalies of congenital Zika; mean head circumference of babies born to mothers exposed during pregnancy, measures of central tendency and dispersion will be presented. For categorical variables, relative frequency distributions will be shown like the proportion of children with adverse outcomes at baseline and subsequent reevaluations among children with microcephaly, with other abnormalities of congenital Zika and children of the cohorts of mothers exposed during pregnancy.

#### 2.8.2. Individual Participant Data Meta-Analyses for Absolute Risks

An Individual Participant Data Meta-Analysis will be undertaken to estimate the absolute risk of having children with congenital Zika manifestations among women infected with ZIKV during pregnancy. To estimate the overall proportion and 95% confidence intervals of congenital Zika manifestations, we will first use a one-stage approach. 

In the one-stage analysis, we will use a hierarchical model with a random effect to account for the clustering of patients within studies. As a sensitivity analysis and to assess the heterogeneity parameter between studies for some key variables, we will also use a two-stage approach. In the two-stage analysis, we will first estimate the study-specific proportions with 95% score confidence intervals. We will then estimate the overall pooled proportion with 95% Wald confidence intervals using the Metaprop command in STATA and investigate heterogeneity between study sites with the I^2^ statistic [25]. 

#### 2.8.3. Individual Participant Data Meta-Analyses for Relative Risks

The comparison of different groups and the introduction of a control group will allow the estimation of the measures of association, i.e., odds ratio and attributable risk. The primary analysis for binary outcomes will be a meta-analysis of one-stage individual data with a random intercept (i.e., considering intra-site correlations) and random effect of ZIKV infection (rather than a fixed effect, therefore considering heterogeneity between sites). This model will use maximum square likelihood. Exposure and outcome variables will be coded as 1/0.

The choice to conduct a one-stage analysis was motivated by the following reasons: rare results (i.e., studies with less than 5 outcome events) may be included; a small number of studies may be included (i.e., addressing situations when a particular outcome has not been measured at all sites); additional adjustments for possible confounders and effect modifiers can be made easily; the exact binomial distribution of data in each study is modeled without correction; and the correlations of intra-study parameters are considered [26].

Odds ratio for the association of ZIKV infection during pregnancy according to the trimester of infection, and congenital Zika manifestations will be estimated using random effects multilevel logistic regression models (i.e., using melogit in STATA). Thus, odds ratios for stillbirths, newborns with microcephaly, brain calcifications in the absence of microcephaly, other abnormalities of central nervous system development and other malformations will also be estimated. The model may be sequentially adjusted for potential confounders. Case definitions will be investigated using sensitivity analyses. Heterogeneity will be investigated using subgroup analyses. Effect modification will be evaluated by the likelihood ratio test.

#### 2.8.4. Meta-Analysis of Two- and One-Stage Individual Data

Associations between maternal ZIKV infection and ongoing continuous outcomes (e.g., head circumference z-scores) will be investigated using both meta-analysis of two- and one-stage individual data. For the two-stage individual participant data meta-analysis approach, we will first fit an analysis of the covariance model to the participants’ individual data from each study site separately. Next, we will perform a random effects meta-analysis of the ANCOVA results from each study using the restricted maximum likelihood estimate. Confidence intervals will be estimated with Hartung–Knapp correction. For the single-stage meta-analysis approach, we will fit random effects ANCOVA (i.e., mixed linear model) with a random intercept (i.e., considering intra-site correlations) and random effects of ZIKV infection. This model will use the restricted maximum likelihood estimate and employ a Kenwood Roger confidence interval correction.

We will also investigate time-to-event outcomes (e.g., time to first seizure) using a study-site stratified one-stage Cox regression model, thus allowing for the study of site-specific baseline risk functions.

All *p*-values will be from two-tailed statistical tests, and all analyses will be performed using Stata, version 15.1 (StataCorp LP, College Station, TX, USA)

#### 2.8.5. Comparison of Subgroups of Children

To characterise the full range of defects and disabilities that may be associated with congenital Zika and their proportional distribution, four groups of children will be compared: (i) children with microcephaly; (ii) children without microcephaly but with symptoms compatible with congenital Zika; (iii) asymptomatic children born to mothers with laboratory evidence of Zika virus infection during pregnancy; and (iv) children born to unexposed mothers.

In the group with microcephaly we will describe all the Zika-related abnormalities and the frequency in which they occur, building the spectrum of the Congenital Zika Syndrome. In the group of children without microcephaly but with symptoms compatible with congenital Zika), we will describe the type of abnormality and their proportional distribution, either isolated or in combination, building the spectrum of congenital Zika in children without microcephaly. The frequency of abnormalities will be compared in these two groups. In the group of children born to women with laboratory evidence of Zika infection during pregnancy but asymptomatic, we will describe the frequency of neurodevelopment delay, which may also be a manifestation of congenital Zika, and compare with the other groups. In the group of children born to unexposed mothers, we will describe the frequency and type of abnormality, if any, and assess their neurodevelopment. This group will be compared with all others, being the standard for the comparisons. Anthropometric measures will also be compared between groups and within groups over time. To estimate the change in head circumference (z-score), weight (z-score) and height (z-score) over time and to estimate the mean difference in Z-scores between clinically relevant subgroups, we will use multilevel mixed effects models accounting for within-child and within-study correlations.

To test the significance of the differences in frequency between groups we will use the Chi-square or the Fisher test, when indicated, and for the comparison of means we will use the F-test or the equivalent nonparametric test.

### 2.9. Ethical Considerations

All participating cohort studies had ethical approval: CAAE 52675616.0.0000.5192; CAAE 53240816.4.0000.5190; CAAE 52803316.8.0000.5192; CAAE 54734316.5.0000.5208; CAAE 52888616.4.0000.5693; CAAE 57792616.1.0000.5030; CAAE 51889315.7.0000.0040; CAAE 65897317.1.0000.5086; CAAE 53611316.0.00005546; CAAE 60168216.2.0000.0005; CAAE 56969516.8.0000.0019; CAAE 68067217.0.0000.0019; CAAE 29124920.6.0000.0019; CAAE 64534017.7.0000.5083; CAAE 56176616.2.1001.5327; CAAE 52675616.0.0000.5269; CAAE 0026.0.009.000-07; CAAE 55465616.0.0000.5275; CAAE 54497216.2.1001.5264; CAAE 56522216.0.0000.5440; CAAE 55805516.2.0000.5415; and CAAE 53248616.2.0000.5412. All participating pregnant women and persons responsible for participating children provided written informed consent form.

## 3. Public Involvement

We held periodical meetings of the principal investigators of the different cohorts to discuss the results of the preliminary analyses and to suggest further points to be explored. Representatives of the Ministry of Health participated in all these meetings and have access to the results and discussions that could inform their guidelines and policies. The final results will also be presented and discussed with local and national health authorities (Secretariats of Health and Ministry of Health) in order to support the adoption of specific public policies aimed at this population.

## 4. ZBC Consortium Governance

With the facilitation and support of the Brazilian Ministry of Health, the governance model of the ZBC Consortium was achieved after agreement between researchers. Briefly, the Executive Secretariat, with regional representation from the research groups, coordinates the consortium and responds directly to the Collegiate, which is composed of representatives from each research group. Under the Collegiate, there is an Analysis Committee that oversees data management and the joint analyses on behalf of the entire Consortium. Members of the Executive Secretariat were elected, and members of the Analysis and Writing Committees were appointed in Collegiate meetings. Representatives of the Ministry of Health (SCTIE, SVS and SAS) are both facilitators and members of the Collegiate. The ZBC Consortium was established based on three principles: decisions are made by consensus between partners, there is no hierarchy between groups, and research groups will lead all initiatives. 

## 5. Discussion and Conclusions

The ZBC Consortium will bring together individual participant data in order to optimize the use of existing epidemiological evidence related to ZIKV infections during pregnancy and congenital Zika manifestations among populations in Brazil. The harmonized database will enable more robust epidemiological analyses that will yield integrated views of this important public health problem. The meta-analyses of individual participant data from the various cohorts in Brazil will create a larger sample size that will facilitate the more precise estimation of congenital Zika manifestations risks and will enable investigation of rare sequelae. In addition, by integrating data from cohorts from different geographic locations and populations across Brazil, this study will enable robust investigations that will improve understanding of the sources of heterogeneity between individual cohort studies. Furthermore, this study will help to overcome challenges arising from the different case definitions used in the published literature to date to identify prenatal ZIKV exposure and congenital Zika-related outcomes. Specifically, this study’s planned sensitivity analyses will make it possible to explore the impact of using different definitions of exposure and categories of outcomes in the estimation of congenital Zika manifestations. In addition to facilitating investigations of established hypotheses, this integrated data platform, with its wealth of information, will also allow the investigators to explore new scientific questions as they arise in the future. The ZBC Consortium will strengthen national partnerships, and the information obtained from this joint study will inform public health policies related to ZIKV. 

We believe that this protocol provides a well-developed, consistent methodology that will be a valuable resource for consistent analysis of disparate sets of data in order to gain a better understanding of the impact of ZIKV infection on pregnant women and their children. This is a reference tool for future studies that are expected to fill in any gaps and presumably refine the methodology. As such, this paper is a resource and will add value in understanding the impact of ZIKV infection during pregnancy. It may also serve as a model to enhance the preparedness of researchers investigating new emerging infectious disease epidemics in the future.

## Data Availability

The data that support the findings of this study are available on the Shared Data Platform, designed and protected by the Informatics Sector of the Aggeu Magalhães Institute/Fiocruz–Pernambuco, Brazil. To guarantee the privacy of all participants’ personal data, access to data is restricted to the coordinators of the ZBC Consortium and, therefore, is not publicly available.
